# Efficient Generation of Somatic Cell Nuclear Transfer-Competent Porcine Cells with Mutated Alleles at Multiple Target Loci by Using CRISPR/Cas9 Combined with Targeted Toxin-Based Selection System

**DOI:** 10.3390/ijms18122610

**Published:** 2017-12-04

**Authors:** Masahiro Sato, Kazuchika Miyoshi, Shingo Nakamura, Masato Ohtsuka, Takayuki Sakurai, Satoshi Watanabe, Hiroaki Kawaguchi, Akihide Tanimoto

**Affiliations:** 1 Section of Gene Expression Regulation, Frontier Science Research Center, Kagoshima University, Kagoshima 890-8544, Japan; 2 Laboratory of Animal Reproduction, Faculty of Agriculture, Kagoshima University, Kagoshima 890-0065, Japan; kmiyoshi@agri.kagoshima-u.ac.jp; 3 Division of Biomedical Engineering, National Defense Medical College Research Institute, Saitama 359-8513, Japan; snaka@ndmc.ac.jp; 4 Division of Basic Medical Science and Molecular Medicine, School of Medicine, Tokai University, Kanagawa 259-1193, Japan; masato@is.icc.u-tokai.ac.jp; 5 The Institute of Medical Sciences, Tokai University, Kanagawa 259-1193, Japan; 6 Basic Research Division for Next-Generation Disease Models and Fundamental Technology, Research Center for Next Generation Medicine, Shinshu University, Nagano 390-8621, Japan; tsakurai@shinshu-u.ac.jp; 7 Animal Genome Research Unit, Division of Animal Science, National Institute of Agrobiological Sciences, Ibaraki 305-8602, Japan; kettle@affrc.go.jp; 8 Department of Hygiene and Health Promotion Medicine, Graduate School of Medical and Dental Sciences, Kagoshima University, Kagoshima 890-0065, Japan; k3038952@kadai.jp; 9 Department of Pathology, Graduate School of Medical and Dental Sciences, Kagoshima University, Kagoshima 890-0065, Japan; akit09@m3.kufm.kagoshima-u.ac.jp

**Keywords:** α-Gal epitope, α-1,3-galactosyltransferase, CRISPR/Cas9, targeted toxin, genome editing, isolectin BS-I-B_4_, LDLR, PTEN, p53

## Abstract

The recent advancement in genome editing such a CRISPR/Cas9 system has enabled isolation of cells with knocked multiple alleles through a one-step transfection. Somatic cell nuclear transfer (SCNT) has been frequently employed as one of the efficient tools for the production of genetically modified (GM) animals. To use GM cells as SCNT donor, efficient isolation of transfectants with mutations at multiple target loci is often required. The methods for the isolation of such GM cells largely rely on the use of drug selection-based approach using selectable genes; however, it is often difficult to isolate cells with mutations at multiple target loci. In this study, we used a novel approach for the efficient isolation of porcine cells with at least two target loci mutations by one-step introduction of CRISPR/Cas9-related components. A single guide (sg) RNA targeted to *GGTA1* gene, involved in the synthesis of cell-surface α-Gal epitope (known as xenogenic antigen), is always a prerequisite. When the transfected cells were reacted with toxin-labeled BS-I-B_4_ isolectin for 2 h at 37 °C to eliminate α-Gal epitope-expressing cells, the surviving clones lacked α-Gal epitope expression and were highly expected to exhibit induced mutations at another target loci. Analysis of these α-Gal epitope-negative surviving cells demonstrated a 100% occurrence of genome editing at target loci. SCNT using these cells as donors resulted in the production of cloned blastocysts with the genotype similar to that of the donor cells used. Thus, this novel system will be useful for SCNT-mediated acquisition of GM cloned piglets, in which multiple target loci may be mutated.

## 1. Introduction

The CRISPR/Cas9-mediated genome editing system is now widely used to obtain cells exhibiting mutations at target loci, after a one-step introduction of DNA or mRNA [1,2]. It involves the co-transfection of a single guide (sg) RNA and Cas9 expression vectors, which include either an antibiotic resistance marker (i.e., puromycin resistance gene) or a fluorescent protein reporter (i.e., green fluorescent protein [GFP]) [3,4,5,6,7,8,9,10]. Upon transfection with these constructs, cells are subjected to selection either in the presence of antibiotics for a short period (3–5 days), or by fluorescence-activated cell sorting (FACS) to enrich genome-edited cells. Antibiotic-based selection is cheaper and more convenient than FACS, but often generates unwanted cells with no mutation in the target locus, probably due to the failure of gene transfer. Furthermore, it may cause occasional chromosomal integration of the markers, Cas9 or sgRNA, which may affect cell survival and function, upon its integration into a locus essential for cellular functions. Hence, drug-free enrichment of the genome-edited cells, through the simple cultivation of cells after transfection with CRISPR/Cas9-related components may be a desirable way to perform cell selection.

We have developed a novel system, named “targeted toxin-based drug-free selection system,” for the enrichment of genetically modified (GM) cells [11,12]. This can be performed by transfecting the cells with an expression vector that can lead to complete suppression of the expression of the α-Gal epitope, one of the carbohydrates expressed on the surface of all mammalian cells, except for humans, and Old-World monkeys [13,14]. In the subsequent steps, the transfected cells are briefly incubated with a solution containing saporin (SAP) toxin-labeled BS-I-B_4_ isolectin (IB4SAP), which shows specific binding to the α-Gal epitope. IB4SAP binds to α-Gal epitope-expressing cells, and kills these cells upon its internalization [15]. The complete suppression of α-Gal epitope expression can be achieved by the forced expression of endo-β-galactosidase C (EndoGalC), an enzyme derived from *Clostridium perfringens*, which cleaves the α-Gal epitope [16,17], or through the inhibition of α-1,3-galactosyltransferase (α-GalT), a key enzyme which is encoded by the *GGTA1* gene, involved in the synthesis of the α-Gal epitope [18,19]. Elimination of the α-Gal epitope from pigs failed to affect their survival; cloned pigs lacking the complete expression of the α-Gal epitope show normal survival [20,21,22,23,24,25]. Thus, α-Gal epitope expression may not be a prerequisite for cell survival and function.

**Figure 1:**
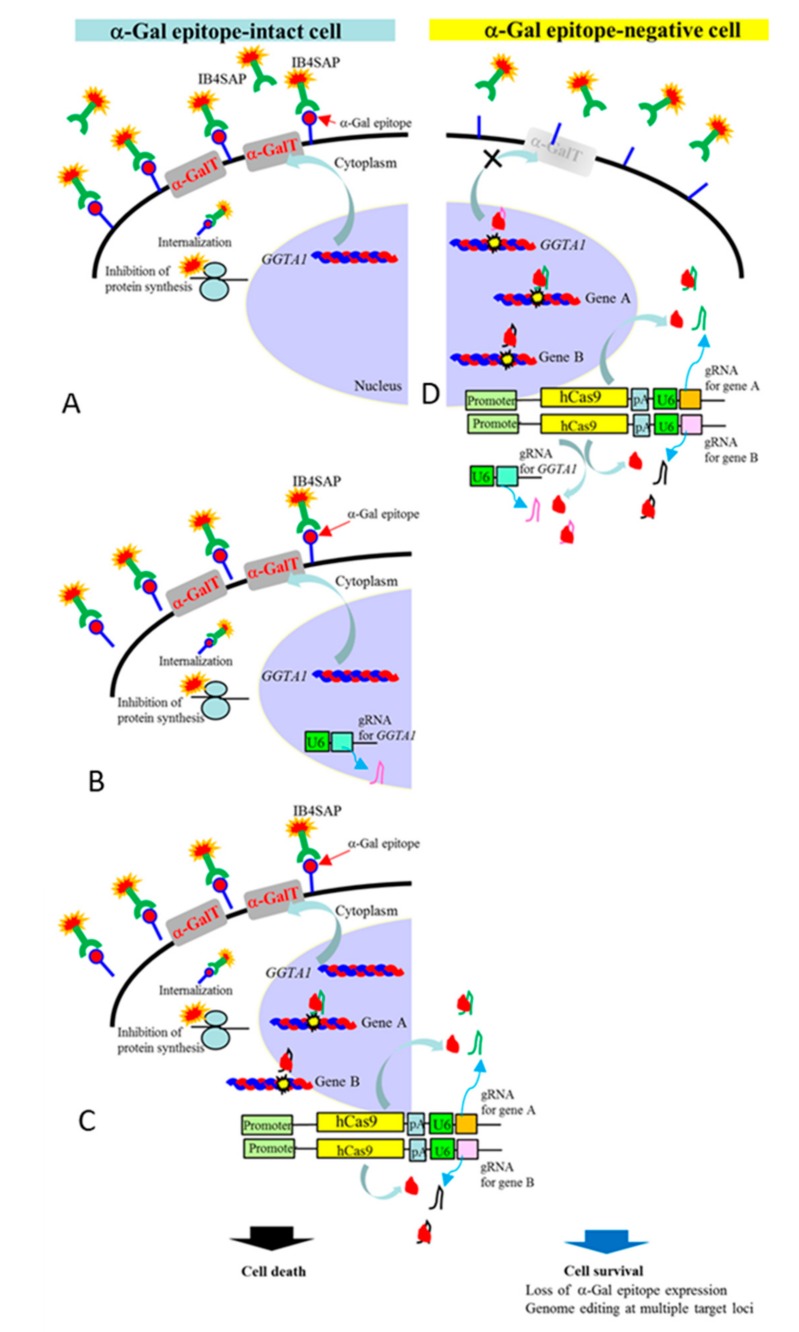
Schematic representation of the enrichment of genome-edited cells using CRISPR/Cas9-based genome editing and targeted toxin technology. Cells were transfected with two pCGsap1-based vectors conferring the expression of both hCas9 and sgRNA (targeted to gene A or B) along with pgRNA#3 vector conferring the expression of sgRNA for *GGTA1*. After 4 days, cells were treated with IB4SAP for a short period. As shown in (**D**), only cells with ablated expression of cell surface α-Gal epitope, due to the disruption in the expression of *GGTA1* that encodes α-GalT, would survive. IB4SAP is known to bind to α-Gal epitope-expressing cells, leading to their death. In these cells, hCas9 from pCGsap1-based vectors and sgRNA targeted to *GGTA1* will be co-expressed, as α-GalT expression has been completely blocked by mutations in *GGTA1*. At the same time, the expression of sgRNAs targeted to genes A and B may occur, leading to the induction of mutations at both loci. However, untransfected cells (shown in (**A**)), cells expressing sgRNA for *GGTA1* (shown in (**B**)) and cells expressing sgRNA for gene A and B (but not *GGTA1*) as well as hCas9 (shown in (**C**)) would undergo cell death, as these cells continue to express α-Gal epitope on their surfaces. U6, U6 promoter; hCas9, humanized Cas9 gene; pA, poly(A) sites.

In this study, we tested whether the targeted toxin-based selection system can be helpful for the efficient enrichment of genome-edited porcine cells. The principle of this system is schematically shown in Figure 1. Porcine cells were transfected with the plasmid pCGsap1, which conferred the expression of both Cas9 and sgRNA (targeted to a gene of interest), and the plasmid pgRNA#3, which conferred the expression of sgRNA targeted to *GGTA1* [19]. Cells carrying both the plasmids would exhibit non-homologous end joining (NHEJ)-based mutations, called insertion-deletion mutations (indels), through the action of Cas9 endonuclease at the target loci [1,2]. Bi-allelic mutations at both alleles for *GGTA1* cause the termination of α-GalT synthesis, leading to the complete loss of α-Gal epitope expression. A brief incubation (37 °C for 2 h) of the transfected cells in the presence of IB4SAP in normal medium promotes the survival of only α-Gal epitope-negative cells. Thus, the surviving cells would lack α-Gal epitope expression, and are highly expected to exhibit induced mutations at other target loci. On the other hand, untransfected cells, and those transfected with pgRNA#3 or pCGsap1 alone, would be eliminated by this treatment, owing to the expression of the α-Gal epitope on their surfaces.

Here, we examined the effectiveness of this novel system for the enrichment of genome-edited porcine cells, and evaluated their applicability for somatic cell nuclear transfer (SCNT)-based production of GM piglets.

## 2. Results

### 2.1. Experiment 1: Genome Editing of Two Target Loci through One-Shot Transfection and Subsequent Selection with IB4SAP

**Figure 2:**
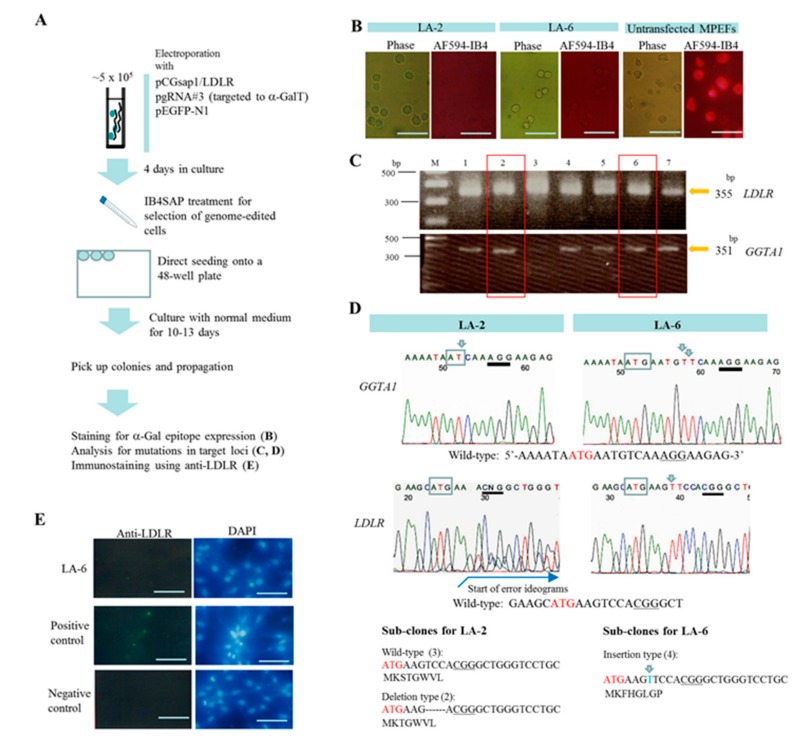
(**A**) Flowchart of the experiments used for testing the feasibility of the new system for enrichment of cells genome-edited at a single target locus. Four days after transfection with pCGsap1/LDLR, pgRNA#3, and pEGFP-N1, cells were treated with IB4SAP for a short period and cultured in normal medium for more than 10 days. The emerging colonies were propagated for molecular biological and cytochemical analyses; (**B**) cytochemical staining of clones LA-2 and -6 with AF594-IB4. Untransfected MPEFs were stained as positive control. Phase, photographs taken under light microscopy; AF594-IB4, photographs taken under UV illumination to detect AF594-IB4–derived red fluorescence. Bar = 30 μm; (**C**) electrophoretic pattern of polymerase chain reaction (PCR) products derived from the amplification of LA-1 to -7. Arrows indicate the size of PCR products for each gene (*LDLR* and* GGTA1*). M, 100 bp-ladder markers; (**D**) direct sequencing of PCR products from LA-2 (left panel) and LA-6 (right panel) using primers specific to *LDLR* or *GGTA1* gene. Arrows above ideograms indicate the sites showing indels. In the bottom of each panel, sequencing results of inserts sub-cloned into TA cloning vector are shown. The numbers of clones examined are shown in parentheses. The translation initiation codon ATG is shown by boxes or in red. PAM is indicated by underlines. The deleted portion in the clones is shown by dotted lines; (**E**) immunocytochemistry of LA-6 and intact MPEFs (used as positive control) using anti-LDLR antibody. The parental MPEFs reactive to the second antibody (fluorescein-labeled anti-mouse IgG) alone are designated as negative control. All cells were counterstained with DAPI upon reaction with the second antibody. Bar = 30 μm.

We first attempted to examine whether the endogenous low-density lipoprotein receptor (*LDLR*) gene undergoes efficient genome editing in cultured porcine cells using our system. *LDLR* mediates the endocytosis of cholesterol-rich LDL, thereby maintaining the plasma level of LDL [26]. Mutations in the gene that encodes the *LDLR* are known to cause familial hypercholesterolemia [27]. The complete suppression of *LDLR* gene expression in pigs is thought to be involved in the pathogenesis of atherosclerosis [28]. Microminipig embryonic fibroblastic cells (MPEFs) were transfected with pCGsap1/LDLR (Table 1), a plasmid that confers the expression of both humanized (h) Cas9 and sgRNA (targeted to the LDLR gene), and the pgRNA#3 plasmid [19], by nucleofection. This was followed by treating the cells with IB4SAP for a short period (Figure 2A). Cells were cultured in a normal medium for more than 10 days, allowing them to grow as independent colonies (Figure 2A). After the selection of the emerging colonies, seven clones (designated as LA-1 to LA-7) were successfully propagated. Staining using Alexa Fluor 594-labeled BS-I-B_4_ isolectin (AF594-IB4) showed negative results for five out of these seven clones (Figure 2B). Performing a polymerase chain reaction (PCR) for the amplification of a region spanning the mutated target gene, using the genomic DNA isolated from these clones, resulted in the successful production of 355- and 351-bp bands of expected size for the *LDLR* and *GGTA1* genes, respectively (Figure 2C). Direct sequencing of the 351-bp PCR products (corresponding to *GGTA1*) revealed that all five α-Gal epitope-negative clones had indels between the start codon (ATG), and protospacer adjacent motif (PAM), creating amino acids that are different from those of the wild-type α-GalT (Table 2). The results for LA-2 and LA-6 are shown as typical examples in Figure 2D. No disordered ideograms downstream of ATG were noted in these samples, indicating that all these samples were bi-allelic knockouts (KOs) for the *GGTA1* locus. However, two other clones (LA-1 and LA-3), which showed a positive result after staining with AF594-IB4, had normal sequences between ATG and the PAM (Table 2). Direct sequencing of the 355-bp PCR products (corresponding to* LDLR*) revealed that all of the α-Gal epitope-negative clones were either mono-allelic (three; LA-2, LA-5, and LA-7), or bi-allelic (two; LA-4 and LA-6) for the mutated *LDLR* alleles (Figure 2D and Table 2). In Figure 2D, the results for the LA-2 and LA-6 clones are shown as typical examples. The LA-2 samples exhibited disordered ideograms downstream of ATG (indicated by the start of error ideograms), indicating the presence of multiple (at least one) mutations. In contrast, no disordered ideograms were noted in LA-6 samples, thereby suggesting the presence of homozygous bi-allelic mutations. In contrast, LA-1 and LA-3, which were α-Gal epitope-positive clones, had normal sequences for the *LDLR* gene (Table 2).

For the detailed determination of the types of mutations present in the LA-2 and LA-6 samples, the 355-bp PCR products were sub-cloned into a TA cloning vector, for sequencing using the *LDLR*-3S primer (Table S1). At least two types of clones were detected for LA-2: wild-type (three clones) and deletion type (two clones), suggesting that LA-2 bears mono-allelic mutations on the *LDLR* alleles (Figure 2D). The amino acid sequence of the wild-type allele was denoted as MKSTGWVL- -, while that of the mutated allele was denoted as MKTGWVL- -, in which only one amino acid (S), the third amino acid from ATG (M), had been deleted. Sub-cloning the LA-6 sample yielded only (four) clones that had single nucleotide insertions at the same position (Figure 2D), indicating that LA-6 has homozygous bi-allelic mutations on the *LDLR* alleles. The amino acid sequence in the mutated allele was denoted as MKFHGLGP- -, and it created an abnormal protein that was different from its wild-type counterpart. Immunocytochemical staining using anti-LDLR antibody showed negative results for the LA-6 clone (Figure 2E).

### 2.2. Experiment 2: Genome Editing of Two Target Loci through One-Shot Transfection and Subsequent Selection with IB4SAP

**Figure 3:**
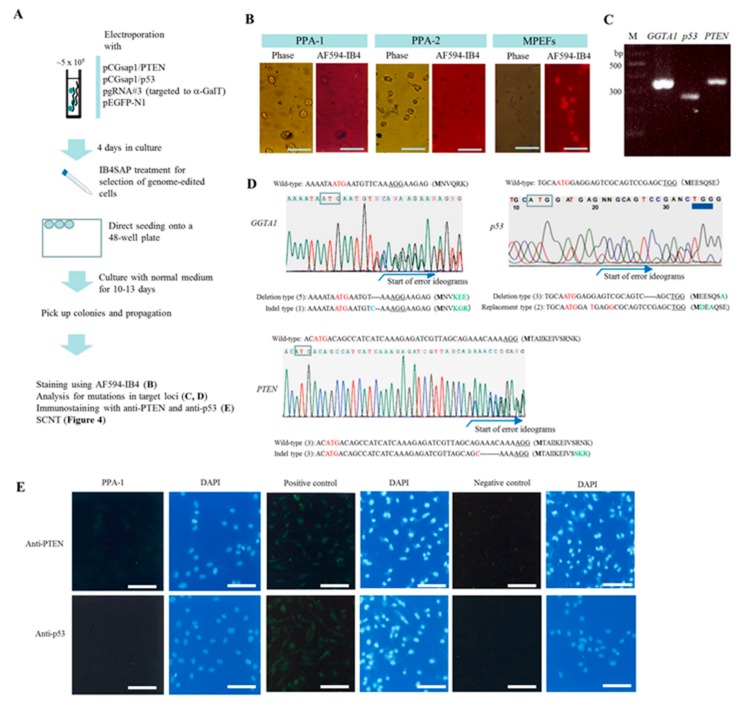
(**A**) Flowchart of the experiments used for testing the feasibility of the new system for the enrichment of cells genome-edited at multiple target loci. Four days after transfection with pCGsap1/PTEN, pCGsap1/p53, pgRNA#3, and pEGFP-N1, cells were treated with IB4SAP for a short period and cultured in normal medium for more than 10 days. The emerging colonies were propagated for molecular biological and cytochemical analyses; (**B**) cytochemical staining of clones PPA-1 and -2 and untransfected MPEFs with AF594-IB4. Phase, photographs taken under light microscopy; AF594-IB4, photographs taken under UV illumination to detect AF594-IB4–derived red fluorescence. Bar = 30 μm; (**C**) electrophoretic pattern of PCR products derived from the amplification of PPA-1 using primer sets specific to each gene (*GGTA1*, *p53*, and *PTEN*). M, 100 bp-ladder markers; (**D**) direct sequencing of PCR products from PPA-1 using primers specific to *PTEN*,* p53*, or *GGTA1* gene. In the bottom of each ideogram, sequencing results of inserts sub-cloned into TA cloning vector are shown. The numbers of clones examined are shown in parentheses. The start codon ATG is shown by boxes or in red. PAM is indicated by underlines. The deleted portion in the clones is shown by dotted lines; (**E**) immunocytochemistry of PPA-1 using anti-PTEN and anti-p53 antibodies. The parental MPEFs are used as positive control. The parental MPEFs reactive to the second antibody (fluorescein-labeled goat anti-rabbit IgG) alone are designated as negative control. All cells were counterstained with DAPI upon reaction with the second antibody. Bar = 30 μm.

We then examined whether the two target loci (*p53* and *PTEN*) can undergo simultaneous genome editing with our system. p53 is a nuclear transcription factor known as a tumor suppressor protein [29]. Phosphatase and tensin homolog deleted on chromosome 10 (*PTEN*) is one of the most commonly lost tumor suppressors in human cancer [30]. In mice, elimination of the expression of both these proteins resulted in cancer cell generation at a high rate [31,32]. For this experiment, we performed pgRNA#3 transfection, IB4SAP treatment, and colony isolation and characterization, as described under Experiment 1, with the exception that two pCGsap1-based vectors (pCGsap1/p53 and pCGsap1/PTEN; Table 1) were used in this case, instead of pCGsap1/LDLR (Figure 3A). After transfection, and the subsequent selection of colonies with IB4SAP, we obtained two clones (termed PPA-1 and PPA-2; Table 3). AF594-IB4 staining showed negative results for both of these clones (Figure 3B), indicating that these clones were bi-allelic KOs for the *GGTA1* locus. To characterize these clones, their genomic DNAs were isolated and subjected to PCR amplification for the region containing indels at the target gene. We successfully obtained the PCR products for each gene. The typical example from the PPA-1 clone is shown in Figure 3C. Direct sequencing of the PCR products from the PPA-1 clone showed disorders in the ideograms for each target gene, indicating the presence of mono-allelic mutations for each gene or mosaics (a mixture of wild-type and bi-allelic KO cells; Figure 3D). For clarification, we sub-cloned these PCR products into the pTA cloning vector. Sequencing analysis of the resulting clones revealed the presence of clones carrying the wild-type insert, and those with indels concerning the *PTEN* gene, at a ratio of 1:1, indicating mono-allelic KOs for the *PTEN* gene (Figure 3D and Table 3). The amino acid sequence in the wild-type allele was denoted as MTAIIKEIVSRNK- -, while that in the mutated allele was denoted as MTAIIKEIVSSKR- -, in which the amino acids R and N, at positions 11 and 12 (counting from the “M”), respectively, were changed to S in the mutated allele, and a stop codon did not appear after S, showing no essential amino acid change in the mutated allele. Clones with two types of indels for the *p53* locus were observed (ratio of 3:2; Figure 3D), and no clone with the wild-type sequence was detected (Figure 3D and Table 3). This suggests the presence of bi-allelic KOs for the *p53* gene. One mutated allele had a deletion type of sequence, coding for MEESQSA- -, which created a premature stop codon at amino acid 27. The other mutated allele had a replacement type of sequence, coding for MDEAQSE- -, in which only amino acids 2 and 4 were different from the wild-type sequence (MEESQSE- -). Clones with one type of indel for the *GGTA1* locus (generating MNVKEE- -, which created a premature stop codon at amino acid 17) were predominant; no clone with the wild-type sequence was noted (Figure 3D and Table 2), indicative of bi-allelic KOs for the *GGTA1* gene. Immunocytochemical staining with the anti-PTEN antibody revealed positive results for PPA-1 cells, suggesting that this clone has mono-allelic KO mutations, as pointed out in the sequence analysis (Figure 3E). In contrast, no reactivity against the anti-p53 antibody was reported (Figure 3E), indicating bi-allelic KOs for the *p53* gene. Similar to these observations, PPA-2 cells showed the following phenotypes: bi-allelic KOs for the *GGTA1* and *p53* genes, and mono-allelic KOs for the *PTEN* genes (Figure 2)

**Figure 4:**
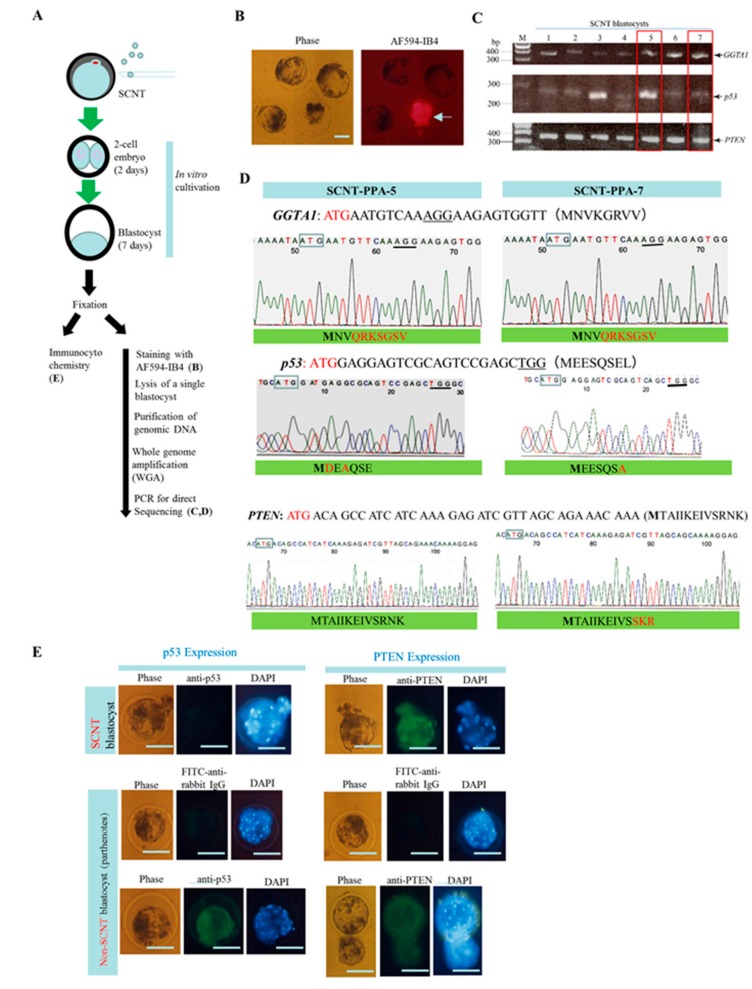
(**A**) Flowchart of somatic cell nuclear transfer (SCNT) experiment using PPA-1 as an SCNT donor. On days 2 and 7 after SCNT, developmental rates of SCNT embryos to two-cell and blastocyst were checked. The blastocysts developed were subjected to AF594-IB4 staining or immunostaining with antibodies. The AF594-IB4-stained blastocysts were used for genomic DNA isolation for molecular biological analyses; (**B**) staining of SCNT blastocysts with AF594-IB4. Phase, photographs taken under light microscopy; AF594-IB4, photographs taken under UV illumination to detect AF594-IB4–derived red fluorescence. Bar = 30 μm; (**C**) electrophoretic pattern of PCR products (SCNT-PPA-1 to 7) derived from a single SCNT blastocyst using primer sets specific to each gene (*GGTA1, p53*, and *PTEN*). The PCR products enclosed by red box were subjected to direct sequencing. M, 100 bp-ladder markers; (**D**) direct sequencing of PCR products from SCNT-PPA-5 (left panel) and SCNT-PPA-7 (right panel) using primers specific to* PTEN*,* p53*, or *GGTA1* gene. The start codon ATG is shown by boxes or in red. PAM is indicated by underlines. Below the ideograms, deduced amino acid sequence is shown. The amino acid sequence shown by red was different from that of the authentic protein; (**E**) immunocytochemistry of SCNT blastocysts using anti-PTEN and anti-p53 antibodies. Parthenogenetically developed blastocysts (shown as non-SCNT blastocyst) were used as positive control. The non-SCNT blastocyst reactive with only second antibody (fluorescein-labeled anti-rabbit IgG) were designated as negative control. All embryos were counterstained with DAPI upon reaction with the second antibody. Bar = 30 μm.

To test whether the phenotype (absence of *p53* and α-GalT expression, but presence of PTEN expression) observed in the PPA-1 clone is transmitted to the cloned blastocysts, SCNT was performed using PPA-1 cells as donors (Figure 4A). The experiments were repeated thrice, and it was seen that between 25.9% and 31.3% of SCNT embryos successfully developed into blastocysts, regardless of the presence or absence of treatment with valproic acid (VPA), after activation by an electric pulse of the SCNT-treated oocytes (Table S2). All of the developing blastocysts were fixed with 4% paraformaldehyde (PFA) for 30 min at 4 °C, and stored at 4 °C prior to cytochemical staining with AF594-IB4, and subsequent molecular biological analysis. In addition, some embryos were subjected to immunocytochemical staining. Almost all (89%; 16/18 tested) of the blastocysts were negative for the α-Gal epitope, as observed with AF594-IB4 staining (Figure 4B). Lectin staining yielded positive results only for a few blastocysts (2/18 tested) (arrow in Figure 4B); these embryos may be derived from cells that escaped and survived IB4SAP treatment, or from oocytes that exhibited parthenogenetic development, following failure of SCNT. In this study, we chose seven AF594-IB4-negative blastocysts, which were subjected to lysis for subsequent genomic DNA isolation. The resulting samples (named as SCNT-PPA-1 to SCNT-PPA-7) were used for whole genome amplification (WGA) [33,34], to amplify the whole genome before PCR analysis. The second-round of PCR of the WGA samples yielded clear bands with the expected size for each target gene (Figure 4C). The PCR products shown in lane 5 (corresponding to SCNT-PPA-5), and 7 (corresponding to SCNT-PPA-7) (Figure 4C) were chosen to examine if these samples display the mutated sequence found in the PPA-1 cells. Direct sequencing of the PCR products demonstrated that both samples exhibited no disorders in the ideograms for the *GGTA1* gene, and shared the mutated sequence of PPA-1 cells. For instance, the putative amino acid sequence of α-GalT was MNVKGRVV- -, while the mutated sequence was MNVQRKSGSV- - (Figure 4D). In the latter case, a stop codon appeared at amino acid 8, showing a premature stoppage of protein synthesis. For the *p53* gene, both samples exhibited no disorder in the ideograms just below ATG, but the sequence was detected in the donor cells (PPA-1 cells) (Figure 3D versus Figure 4D); it differed from the wild-type sequence, leading to frameshift of p53 amino acid (Figure 4D). Thus, both SCNT-PPA-5 and SCNT-PPA-7 are deemed as bi-allelic KO mutants for the *p53* gene (MDEAQSE- - for SCNT-PPA-5 and MEESQSA- - for SCNT-PPA-7). These mutated alleles are also observed in the donor cell PPA-1 (Figure 3D versus Figure 4D). With respect to the *PTEN* gene, both SCNT-PPA-5 and SCNT-PPA-7 exhibited no disorder in the ideograms, as seen after direct sequencing. Thus, SCNT-PPA-5 and SCNT-PPA-7 were deemed as wild-type (+/+; MTAIIKEIVSRNK- -), and bi-allelic KO (MTAIIKEIVSSKR- -) clones, respectively, for the *PTEN* gene. The mutated allele found in SCNT-PPA-7 was also observed in the PPA-1 cells (Figure 3D versus Figure 4D).

We performed immunocytochemical staining of SCNT blastocysts using anti-PTEN and p53 antibodies to confirm the results observed in molecular biological analysis. All test samples (6/6) and the negative control parthenogenetic blastocysts showed no reaction against anti-p53. On the other hand, the positive control parthenogenetic blastocysts exhibited a strong reaction with anti-p53 (Figure 4E). In contrast, all of the embryos tested (8/8) exhibited positive reaction with anti-PTEN similar to that observed with the positive control parthenogenetic blastocysts. The negative control parthenogenetic blastocysts reacted only with the second antibody (Figure 4E).

### 2.3. Experiment 3: Detecting Possible Off-Target Mutations in the Target Sites of Genome-Edited Cells

It is known that CRISPR/Cas9-based genome editing often causes off-target mutations in irrelevant loci in certain cell types [35,36]. To potentially identify mutations induced at off**-**target sites, we selected three candidate genes as potential off-target sites, based on the prediction method published in (Available online: http://crispr.mit.edu). These genes are the top 3 high-scoring genes (ranging from 3.3 to 1.1; Table S4). For the T7 endonuclease I (T7E1) assay, we used the LA-6 clone that is judged as a bi-allelic KO for the *LDLR* gene, and the PPA-1 clone that is judged as a bi-allelic KO for the *p53* gene, and a mixture of cells with bi-allelic KO for the *PTEN* gene and wild-type cells, together with normal intact MPEFs as a control. A T7E1 assay demonstrated that no off-target cleavage was detected in the samples examined, except for the #1 PTEN sample which showed cleaved bands when the mixture of PPA-1- and MPEF-derived products was subjected to digestion with T7E1 (Supplementary Figure S2). 

## 3. Discussion

The aim of this study was to obtain porcine cells having indels at target loci by efficient one-shot co-transfection of CRISPR/Cas9-related plasmid DNA; there are several methods to isolate cells that have been successfully genome-edited in vitro. For example, the use of selective drugs, such as G418 and puromycin, and FACS has been frequent [3,4,5,6,7,8,9,10]. In the case of the former approach, after transfection, the cells must be incubated in a medium containing a selective drug for several days, to remove the untransfected cells. However, it often causes occasional chromosomal integration of transgenes, which may affect cellular survival and function, probably due to the integration taking place in a locus essential for cell function. Furthermore, transient treatment with these drugs often allows some untransfected cells to survive, which may decrease the efficiency of the generation of the genome-edited cells. The latter approach requires an expensive machine to sort cells, and manpower to operate this machine. Fujimura et al. [37] first demonstrated the usefulness of magnetic-activated cell sorting-based selection of α-Gal epitope-negative cells. The α-Gal epitope-positive cells bound to biotin-labeled IB4 could be successfully eliminated. Since then, several laboratories have successfully isolated genome-edited α-Gal epitope-negative cells using this system [24,25,37,38,39,40,41,42]. It is noteworthy that most previous study reports were confined only to the isolation of *GGTA1* KO cells, except for the report by Li et al. [40], who successfully isolated α-Gal epitope-negative cells with multiple genetic modifications.

In this study, we employed a targeted toxin-based technology to obtain α-Gal epitope-negative clones, which is similar to the aforementioned magnetic bead-based selection system, but depends on the isolation of *GGTA1* KO cells that survive after the death of α-Gal epitope-positive cells, as shown in Figure 1. With this system, it is possible to isolate cells lacking endogenous *GGTA1* gene expression, via the co-expression of an sgRNA expression vector (pgRNA#3; targeted to* GGTA1*), and the hCas9 protein, obtained from pCGsap1-based vectors (pCGsap1/LDLR or pCGsap1/PTEN and pCGsap1/p53). At this time, indels at one (*LDLR*) or two (*p53* and *PTEN*) target loci were expected to be simultaneously induced in these clones, owing to the co-introduction of pCGsap1-based vectors upon transfection (see Figure 1). Following co-transfection with pCGsap1/LDLR and pgRNA#3, a total of seven clones were isolated, of which, two (LA-1 and LA-3) expressed the α-Gal epitope and therefore, had the normal *LDLR* gene. However, the remaining five α-Gal epitope-negative clones had all indels in the *LDLR* locus (Table 2). Furthermore, co-transfection with pCGsap1/PTEN, pCGsap1/p53, and pgRNA#3 resulted in two clones that had indels at each of the two target loci (Table 3). Thus, our strategy to obtain cells with indels at a higher efficiency appears to be feasible, and highly effective.

**Figure 5:**
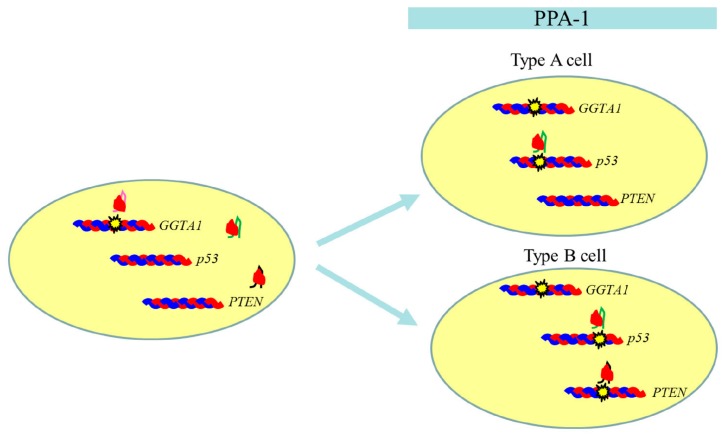
Possible mechanism underlying generation of two types of cells in PPA-1. When a cell is simultaneously transfected with three types of vectors (pCGsap1/PTEN, pCGsap1/p53, and pgRNA#3), hCas9/sgRNA (for* GGTA1*) complex is first transferred to *GGTA1* to induce bi-allelic indels, thereby causing dysfunction of α-GalT. After cell division, two α-Gal epitope-negative daughter cells are generated. In one daughter cell, called type A, hCas9/sgRNA (for p53) complex attacks the target locus for the generation of bi-allelic indels, while *PTEN* locus remains intact. In the other daughter cell, called type B, hCas9/sgRNA (for p53) creates bi-allelic indels on a region different from that in type A cell, while *PTEN* locus is genome-edited by hCas9/sgRNA (for PTEN) to create bi-allelic indels.

Our final goal was to obtain GM piglets that were KOs for multiple target loci. For this purpose, the use of GM porcine cells with a high degree of purity was a prerequisite. However, the present SCNT-based experiment revealed that the donor cells used for SCNT may be a mixture of cells harboring different mutations at the target sequence. For instance, at least two types of cells appeared to exist in the PPA-1 clone. One of these cell types (type A) had bi-allelic mutations at the *GGTA1* and p53 loci, but a wild-type sequence at the *PTEN* locus. The other cell (type B) displayed bi-allelic mutations at the *GGTA1* (with the same mutation seen in type A cell), p53 (with mutations at the portion different from that found in type A cell), and *PTEN* loci (Figure 5). The mechanism involved in the generation of such variations in the PPA-1 clone is questionable. As shown in Figure 5, the bi-allelic mutation may have first occurred in the *GGTA1* locus, following binding of the hCas9/sgRNA (obtained from pgRNA#3 vector) complex to a DNA target. As a consequence, mutations targeted to the p53 and PTEN loci may be transferred to its daughter cells. There may be a difference in the accessibility towards the target loci among the Cas9/sgRNA complexes. Hence, a careful selection of clones deemed suitable for SCNT is needed.

It is noteworthy that off-target effects are also a serious concern when utilizing a CRISPR/Cas9 system for inducing mutations at a target locus. In our previous study, we assessed this problem, and found that the microinjection of CRISPR/Cas9-related mRNA into parthenogenetic porcine embryos resulted in successful genome editing at the target locus (i.e., *GGTA1* coding for α-GalT), but did not induce any mutation in the other genes [43]. In the present study, we also tested for this possibility in the target loci (i.e., *LDLR*, *p53* and *PTEN*), and found no off**-**target mutations in these sites, except for the #1 PTEN site which showed off**-**target mutations (see Supplementary Figure S2). 

As noted previously, there is no report that shows that GGTA1 has an essential role in embryogenesis, and the growth/function of the mutants, or that *GGTA1* mutations induce the death of cloned animals. However, relatively high abortion rates and low birth weights were observed [21,39,44]. According to Cheng et al. [41], these abnormal phenotypes may be partly due to the incomplete reprogramming of somatic cells during the process of SCNT. 

It is interesting that all the SCNT embryos tested were unreactive to anti-p53 (Figure 4E), despite SCNT-PPA-5 having two amino acid changes at the N-terminus of p53 (see Figure 4D). This is in contrast with the case of PTEN, because all SCNT embryos tested expressed PTEN, as shown in Figure 4E. As previously mentioned, PPA-1 clone should comprise cells which are bi-allelic KOs for PTEN, similar to the previously mentioned type B cells (see Figure 5). Therefore, some of these SCNT blastocysts may be unreactive to the anti-PTEN antibody. However, the fact that PPA-1 cells was reactive to the anti-PTEN antibody (see Figure 3E) suggests that the PTEN protein produced from PPA-1 cells was functional. When we checked the mutated allele for PTEN in PPA-1 cells, the PTEN N-terminal portion was only changed from RN to S (see Figure 3D), which failed to cause a frameshift mutation. This slight amino acid change would not have affected the reactivity of PPA-1 cells, and that of SCNT blastocysts to anti-PTEN. The most promising way to avoid such subtle mutations caused by indels may be a CRISPR/Cas9-mediated induction of large deletions (spanning a few exons, including ATG) in the *PTEN* sequence.

## 4. Materials and Methods 

### 4.1. Cells

MPEFs derived from a 30-day-old fetus (named FFs 4 in the previous study [45]) were used for transfection experiments, as these cells exhibited relatively good proliferation. These were maintained in vitro using porcine embryonic fibroblast (PEF) medium, according to the conditions previously described [46,47]. For each experiment, cells passaged for 4–8 generations were used.

The experiments described were performed in agreement with the guidelines of Kagoshima University Committee on Recombinant DNA Security and approved by the Animal Care and Experimentation Committee of Kagoshima University (No. S28003; dated on 16 May 2016).

### 4.2. Construction of pCGsap1-Based Vectors for Simultaneous Expression of hCas9 and sgRNA

A fragment containing U6 promoter and sgRNA targeted to enhanced green fluorescent protein (EGFP) was amplified with PCR using gRNA_GFP-T1 vector (#41819; Addgene; available online: https://www.addgene.org/41819/) as a template. *Xho*I and *Eco*RI restriction sites were placed at both ends. The resulting PCR products were cloned between *Xho*I and *Eco*RI sites of pBluescript SK(-) (Stratagene, La Jola, San Diego, CA, USA) to create pBSK-GFPT1. Next, the fragment containing GFPT1 in pBSK-GFPT1 was replaced by *Sap*I adaptor linker (5′-GAAGAGCCTCGAGGAATTCGCTCTTC-3′) to create pGsap1. Furthermore, a *Not*I-digested fragment containing CAG promoter (comprising cytomegalovirus enhancer and chicken β-actin promoter), NFLCas9 gene (carrying nuclear location signal + FLAG tag in front of hCas9 gene), and poly(A) sites isolated from pCAG-NFLCas9pA [48] was inserted into the *Not*I site of pGsap1. The resulting plasmid was termed as pCGsap1 (Supplementary Figure S1).

The oligonucleotides targeted to *LDLR*,* PTEN*, or *p53* genes (Table 1) obtained from an outside order were inserted into the *Sap*I site in pCGsap1 vector to obtain pCGsap1/LDLR, pCGsap1/PTEN, and pCGsap1/p53, which can confer simultaneous expression of hCas9 and sgRNA for *LDLR*,* PTEN*, and *p53* genes, respectively. The fidelity of the inserted sgRNA was confirmed by sequencing. To ablate the expression of α-GalT, pgRNA#3 [19] that contains sgRNA targeted to *GGTA1* was used. pEGFP-N1 plasmid (Invitrogen Co., Carlsbad, CA, USA) was used as a maker for monitoring the successful transfection upon co-transfection. 

### 4.3. Transfection and Selection of Genome-Edited Clones after Targeted Toxin-Based Selection

The trypsinized MPEFs (5 × 10^5^ cells) were mixed with 90 μL of electroporation buffer (Ingenio solution; # MIR 50111; Mirus Bio LLC, Madison, WI, USA) containing 3 μg of pCGsap1 vectors (both pCGsap1/PTEN and pCGsap1/p53 or pCGsap1/LDLR alone), 3 μg pgRNA#3, and 1 μg pEGFP-N1 and subjected to electroporation using Lonza Nucleofector system under B-024 condition [46]. After electroporation, cells were seeded onto a gelatin-coated 60-mm dish (#4010-020; Iwaki Glass Co. Ltd., Tokyo, Japan) containing 3 mL of normal PEF medium. Four days after culture, the cells were trypsinized and the aliquots (~10^5^ cells) were subjected to treatment with 80 μg/mL IB4SAP (#IT-10; Advanced Targeting Systems Inc., San Diego, CA, USA) in 20 μL of Dulbecco’s modified phosphate-buffered saline without Ca^2+^ and Mg^2+^, pH 7.4 (hereinafter referred to as D-PBS) containing 2% fetal bovine serum (FBS) and 1 mM calcium chloride (CaCl_2_; hereafter referred to as PBS/FBS/CaCl_2_) at 37 °C for 2 h, according to the procedure described by Akasaka et al. [11] and Sato et al. [12]. The remaining cells were deep-frozen in CELLBANKER^®^ 1, a serum-containing cryopreservation medium (#CB011; TaKaRa Bio Company, Shiga, Japan) for future use. IB4SAP-treated cells were seeded onto a 60-mm gelatin-coated dish containing 3 mL PEF medium supplemented with 0.1 mM ascorbic acid (#074-04581; Wako Pure Chemical Industries Ltd., Tokyo, Japan) and 10 ng/mL of basic fibroblast growth factor (#354060; BD Biosciences, San Jose, CA, USA) and cultured for 10–13 days to allow generation of viable colonies. The resultant colonies were marked at the bottom of the dish, washed with 2 mL of a solution containing 0.125% trypsin and accutase (#AT104; Innovative Cell Technologies, Inc., San Diego, CA, USA) at a ratio of 1:1 (*v*/*v*), and allowed to dissociate by brief incubation (5 min at 37 °C). The colonies were isolated using a Gilson Yellow pipette tip (200 μL). The isolated cells were placed in normal PEF medium (~500 μL) in the wells of gelatin-coated 48-well plate (#380-048; Iwaki Glass Co. Ltd.) and subjected to step-wise propagation. During this process, some cells were subjected to staining with AF594-IB4 (2 μg/mL; #I21413; Invitrogen Co.) in PBS/FBS/CaCl_2_. The remaining cells were either frozen or subjected to genomic DNA isolation for the detection of mutated alleles at the target loci. 

### 4.4. SCNT

Somatic cell nuclear transfer was performed according to the method of Sato et al. [49] and Miyoshi et al. [50,51]. Each nucleus from the genome-edited MPEF clones was introduced into a single enucleated oocyte using membrane fusion by applying a single direct-current pulse. Some fused embryos were treated with 8 mM VPA for 24 h after activation because this treatment improved the in vitro development of microminipig SCNT embryos in our previous study [45]. Blastocysts derived from parthenogenetically activated embryos were used as control, according to the previously described method [52]. The development of SCNT embryos was evaluated by the rates of cleavage and blastocyst formation at two and seven days of culture, respectively. The developing blastocysts were fixed in 4% PFA in D-PBS for 30 min at room temperature and stored at 4 °C in D-PBS covered with paraffin oil in a Terasaki microtest plate (Nunc, Roskilde, Denmark) prior to molecular biological analysis.

### 4.5. Genomic DNA Isolation, PCR, and Sanger Sequencing of Mutated Sites 

Genomic DNA was extracted by adding 100 μL of lysis buffer (0.125 μg/mL of proteinase K, 0.125 μg/mL of Pronase E, 0.32 M sucrose, 10 mM Tris-HCl (pH 7.5), 5 mM magnesium chloride (MgCl_2_), and 1% (*v*/*v*) Triton X-100) to the cell pellets (ranging from 10^3^ to 10^4^ cells/mL), followed by vigorous overnight shaking at 37 °C and extraction with phenol/chloroform [19]. The supernatant was precipitated with isopropanol and the precipitated DNA was dissolved in 20 μL of sterile water. The DNA was stored at 4 °C.

The resulting genomic DNA samples (1 μL; approximately 5 ng) were subjected to first PCR using primer sets for each target gene (Supplementary Table S1) in 20 μL volume using the PCR conditions previously described [43]. The expected band sizes of the resulting PCR products are shown in Supplementary Table S1. In some cases, a second round of PCR (nested PCR) was performed using 1 μL of the first PCR product as a template. The PCR conditions used were the same as the first PCR except for the primer sets (Supplementary Table S1). These resulting products (1 μL) were evaluated by loading on a 2% agarose gel. The remaining products were subjected to column-based purification using MACHEREY-NAGEL PCR Clean-Up and Gel Extraction Kit (#U0609B; TaKaRa Bio Company) and finally re-suspended in ~30 μL of sterile water. The DNA content was measured using a spectrophotometer. 

For sequencing of the PCR products, the column-purified PCR products were subjected to direct sequencing at Eurofins Genomics Inc. (Tokyo, Japan). In some cases, PCR products were sub-cloned into a TA cloning vector pCR2.1 (Invitrogen Co.) prior to sequencing.

### 4.6. WGA Using A Single Blastocyst

The fixed single blastocyst was transferred to a PBS drop (1 μL) in a 0.5 mL PCR tube (#PCR-05-C.; AxyGen Scientific, Inc., Union City, CA, USA) with the help of a mouth piece-controlled micropipette, as previously described [33,34]. Genomic DNA was extracted by adding 100 μL of lysis buffer mentioned above and lysed at 37 °C for over two days. The solution was extracted with phenol/chloroform [43] and the supernatant was precipitated with isopropanol. The precipitated DNA was dissolved in 20 μL of sterile water and stored at 4 °C. To increase the amount of whole genomic DNA, we employed WGA using illustra GenomiPhi V2 DNA Amplification Kit (#25-6600-31; GE Health Care Japan, Tokyo, Japan), as previously described [34,43]. Briefly, 2 μL of genomic DNA was mixed with 8 μL reaction buffer containing enzyme in a 20 μL volume and allowed to react overnight at 30 °C. The resulting WGA products (2 μL) were subjected to the first PCR as described above. 

### 4.7. Screening and Detection of Off-Target Sites

The potential off-target sites were identified based on the internet web URL (Available online: http://crispr.mit.edu). From the candidates with the highest scores, three genes were selected (Supplementary Table S4). In order to amplify these genes, the genomic DNA (1 μL) derived from the LA-6 and PPA-1 clones, and MPEFs were first PCR-amplified using different primer sets (Supplementary Table S3) in a reaction volume of 20 μL, using the PCR conditions described above. A nested PCR was then performed, using the products from the first PCR (1 μL), and different primer sets (Supplementary Table S3) in a reaction volume of 20 μL, using the same conditions as the first PCR. After analyzing a portion of the nested PCR products, they were ethanol-precipitated and, finally, dissolved in 20 μL of sterile water. Then, the T7E1 assay was carried out as described below, to confirm whether off-target mutations existed.

### 4.8. T7E1-Based Assay and Sequencing 

For the T7E1-based cleavage assay, 10 μL of 1× NEB2 reaction buffer (New England BioLabs Japan Inc., Tokyo, Japan), containing 400 ng of the nested PCR products derived from the experimental sample (200 ng derived from LA-6 cells and PPA-1 clone), and control sample (200 ng derived from normal PEFs) were placed in a 0.5 mL PCR tube (AxyGen Scientific, Inc.). The PCR tube was incubated at 95 °C for 5 min in a thermal cycler (PC-708; Astec, Fukuoka, Japan), to denature the sample, and then incubated for 0.5–1 h at room temperature (24 °C) to allow for re-annealing and the generation of heteroduplex DNA. 1 μL of T7E1 (2.5 U/μL; New England BioLabs Japan Inc.) was then added to the denatured/re-annealed sample, and the PCR tube was incubated at 37 °C for 1 h. The nuclease-treated solutions were electrophoresed on a 2% agarose gel, and the gel was stained with ethidium bromide, and photographed under ultraviolet illumination.

### 4.9. Staining with AF594-IB4 and Detection of Fluorescence

Staining of cells/embryos with AF594-IB4 was performed as described in our previous study [43]. The stained embryos were examined under a fluorescence microscope (BX60; Olympus, Tokyo, Japan) using DM505 (BP460-490 and BA510IF; Olympus) and DM600 filters (BP545-580 and BA6101F; Olympus), which were used for the detection of EGFP-derived green fluorescence and AF594-derived red fluorescence, respectively. Micrographs were taken using a digital camera (FUJIX HC-300/OL; Fujifilm Co., Tokyo, Japan) attached to the fluorescence microscope and images were printed using a Mitsubishi digital color printer (CP700DSA; Mitsubishi, Tokyo, Japan).

### 4.10. Immunocytochemistry 

For immunocytochemical staining, cells or single blastocysts fixed with 4% PFA in D-PBS were stored in D-PBS containing 2% normal goat serum (PBS-NGS) at 4 °C. These were stained overnight with primary antibodies specific for LDLR (#NBP1-78159; 1:200; Novus Biologicals, LLC, Littleton, CO, USA) at 4 °C. In some cases, fixed cells or single blastocysts were first permeabilized with 0.05% Triton X-100 (Sigma-Aldrich Co. Ltd., Irvine, UK) for 30 min at room temperature and stored in PBS-NGS at 4 °C. The samples were stained overnight with primary antibodies specific for PTEN (#GTX101025; 1:200; Gene Tex Inc., Irvine, CA, USA) or p53 (#sc-6243; 1:200; Santa Cruz Biotechnology, Inc., Santa Cruz, CA, USA) at 4 °C. After washing with PBS-NGS, these specimens were treated overnight at 4 °C with 4′,6-diamidino-2-phenylindole, dihydrochloride (DAPI) (5 μg/mL final concentration; Thermo Fisher Scientific, Inc., Waltham, MA, USA) and the following secondary antibodies: fluorescein-conjugated goat anti-rabbit IgG (H + L) (#FI-1000; 1:200; Vector Laboratories, Burlingame, CA, USA) for anti-PTEN and anti-p53 antibodies and fluorescein-conjugated goat anti-mouse IgG (#BET-2901-16; 1:200; Funakoshi Co., Ltd., Tokyo, Japan) for anti-LDLR antibody. As a negative control, the specimens were overnight incubated with the secondary antibody alone at 4 °C. 

## 5. Conclusions

We developed a novel system for the enrichment of genome-edited cells by co-transfection of pCGsap1-based vectors conferring simultaneous expression of hCas9 and sgRNA (targeted to the target loci) and a vector conferring expression of sgRNA targeted to *GGTA1*, followed by their treatment with IB4SAP. Using this system, we successfully obtained genome-edited porcine cells with one or two target loci displaying mono- or bi-allelic mutations. SCNT using these cells as donors resulted in the acquisition of cloned blastocysts having the mutated sequence of the donor cells at their target loci. Thus, our present method will be useful for the production of cloned GM piglets exhibiting induced mutations at multiple target loci.

## Figures and Tables

**Table 1:** Information on sgRNA used for the construction of pCGsap1/LDLR, pCGsap1/p53, and pCGsap1/PTEN.

Oligonucleotides (Oligo) Used for Cloning into *Sap*I Site in pCGSap1	Target Gene	Reference
*LDLR*-oligo: 5′-ACC GGCTG GAAGC **ATG**AA GTCCA G-3′ 3′-CCGAC CTTCG TACTT CAGGT CAAA-5′	Porcine *LDLR*	*Sus scrofa* breed mixed chromosome 2, Sscrofa10.2 Sequence ID: ref (NC_010444.3)
*p53*-oligo: 5′-ACC GGAGG AGTCG CAGTC CGAGC G-3′ 3′-CCTCC TCAGC GTCAG GCTCG CAAA-5′	Porcine *p53*	*Sus scrofa* breed mixed chromosome 12, Sscrofa10.2 Sequence ID: ref (NC_010454.3)
*PTEN*-oligo: 5′-ACC AGATC GTTAG CAGAA ACAAA G-3′ 3′-TCTAG CAATC GTCTT TGTTT CAAA-5′	Porcine *PTEN*	*Sus scrofa* breed mixed chromosome 14, Sscrofa10.2 Sequence ID: ref (NC_010456.4)

The underline beneath the oligonucleotide indicates sgRNA sequence immediately upstream of PAM. The translation initiation codon ATG is shown in bold.

**Table 2:** Characterization of microminipig embryonic fibroblastic cells (MPEF) clones, LA-1 to 7, after transfection and subsequent selection with IB4SAP.

Property	Loci Targeted	LA-1	LA-2	LA-3	Clones LA-4	LA-5	LA-6	LA-7
Direct sequencing of PCR products ^1^	*GGTA1 LDLR*	Normal Normal	Bi-allelic Mono-allelic	Normal Normal	Bi-allelic Bi-allelic	Bi-allelic Mono-allelic	Bi-allelic Bi-allelic	Bi-allelic Mono-allelic
Expression of α-Gal epitope, as evaluated by cytochemical staining with AF594-IB4 ^2^	*GGTA1*	++	−	++	−	−	−	−
Expression of LDLR, as evaluated by immunocytochemical staining using anti-LDLR ^2^	*LDLR*	+	NT	NT	NT	NT	−	NT

^1^ Genotyping of clones was determined by direct sequencing of PCR products and sometimes by sequencing of inserts sub-cloned into TA cloning vector. ^2^ Fluorescence intensity was expressed as ++ (strong), + (moderate), +/− (slight) or − (none). NT: not tested.

**Table 3:** Characterization of MPEF clones, PPA-1 and -2, after transfection and subsequent selection with IB4SAP.

Clones	Locus	Genotyping of Clones by Direct Sequencing of PCR Products and Inserts Sub-Cloned into TA Cloning Vector	Expression of α-Gal Epitope, as Evaluated by Cytochemical Staining with AF594-IB4	Expression of Protein, as Evaluated by Immunocytochemical Staining Using Antibodies
PPA-1	*GGTA1*	Deletion type (bi-allelic)		−
	*PTEN*	Mixture of indel type (bi-allelic) and wild-type	No	Yes
	*p53*	Mixture of deletion (bi-allelic) and replacement (bi-allelic) types		No
PPA-2	*GGTA1*	Deletion type (bi-allelic)		−
	*PTEN*	Deletion type (mono-allelic)	No	Yes
	*p53*	Insertion type (bi-allelic)		No

## Supplementary Materials

Supplementary materials can be found at www.mdpi.com/1422-0067/18/12/2610/s1.

## Abbreviations

AF594-IB4	Alexa Fluor 594-labeled BS-I-B_4_ isolectin
α-GalT	α-1,3-galactosyltransferase
DAPI	4′,6-Diamidino-2-phenylindole, dihydrochloride
D-PBS	Dulbecco’s modified phosphate-buffered saline without Ca^2+^ and Mg^2+^, pH 7.4
EGFP	Enhanced green fluorescent protein
EndoGalC	Endo-β-galactosidase C
FACS	Fluorescence-activated cell sorting
FBS	Fetal bovine serum
GFP	Green fluorescent protein
GM	Genetically modified
h	Humanized
IB4SAP	Saporin toxin-labeled BS-I-B_4_ isolectin
KO	Knockout
LDLR	Low density lipoprotein receptor
MPEF	Microminipig embryonic fibroblastic cell
NGS	Normal goat serum
NHEJ	Non-homologous end joining
PEF	Porcine embryonic fibroblastic cell
PAM	Protospacer adjacent motif
PCR	Polymerase chain reaction
PFA	Paraformaldehyde
PTEN	Deletion of phosphatase and tensin homolog from chromosome 10
SCNT	Somatic cell nuclear transfer
sg	Single guide
VPA	Valproic acid
WGA	Whole genome amplification
