# Supplementary material for: Efficient Generation of Somatic Cell Nuclear Transfer-Competent Porcine Cells with Mutated Alleles at Multiple Target Loci by Using CRISPR/Cas9 Combined with Targeted Toxin-Based Selection System
Source: Int J Mol Sci. 2017 Dec 4;18(12):2610. doi: 10.3390/ijms18122610 (PMC5751213; doi:10.3390/ijms18122610)
Supplement: Supplementary file 1 [file ijms-18-02610-s001.pdf]

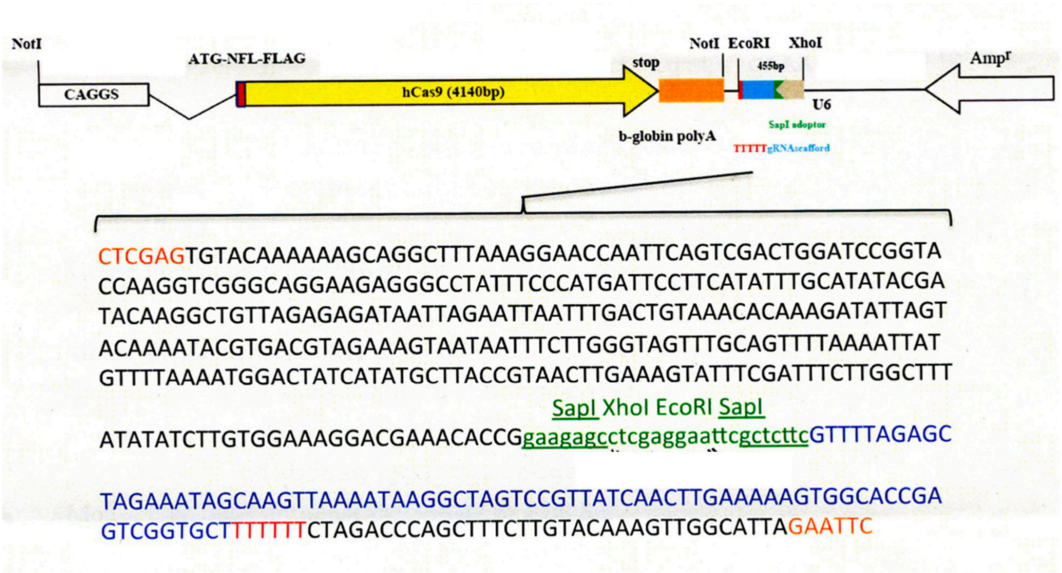

Supplementary Figure 1. Structure of pCGsap1.

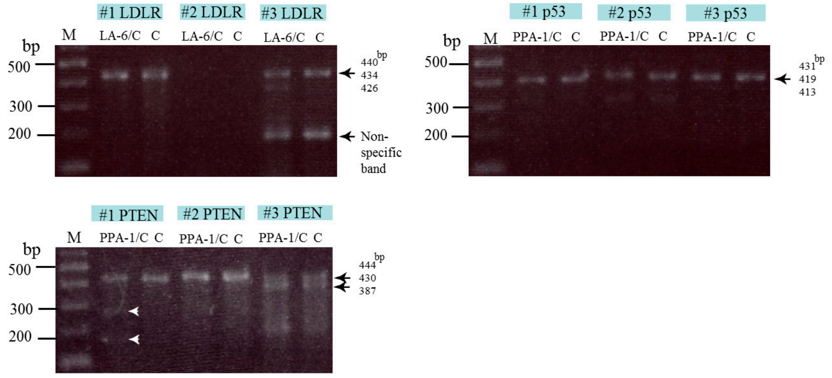

Supplementary Figure 2. T7E1 analyses of three potential off-target sites in LA-6 clones (for *LDLR* gene) and PPA-1 clones (for both *p53* and *PTEN* genes), together with untransfected MPEFs. #1 LDLR, #2 LDLR and #3 LDLR are the potential off-target sites for the *LDLR* gene, with the highest scores (Supplementary Table 4). Similarly, #1 p53, #2 p53 and #3 p53, and #1 PTEN, #2 PTEN and #3 PTEN are potential off-target sites for the *p53* and *PTEN* genes, respectively (Supplementary Table 4). In each case, an approximately 400 bp long band is predominantly observed when no cleavage occurs. If T7E1-mediated cleavage occurs, two bands (~150 and ~250 bp) are expected to be released from the ~ 400 bp band. Unfortunately, the amplification of the ~ 400 bp products failed when we tried to amplify #2 LDLR, in both the LA-6 cells, and the MPEFs. Notably, there is no appreciable off-target cleavage in each of the samples that were amplified successfully, although only the #1 PTEN sample exhibited off-target mutations, as evidenced by the presence of two cleaved bands (shown by arrowheads). **M**, 100 bp ladder markers; LA-6/C, a mixture of LA-6 DNA and MPEF DNA; C, MPEF DNA; PPA-1/C, a mixture of PPA-1 DNA and MPEF DNA.

Supplementary Table 1. Primers used for PCR-based amplification of target sequences.

Primer sets	Sequence (5′-3′)	Purpose	Expected size (bp)	Locus	Reference
Ex4-S<br>Ex4-RV	GCAAATTAAGGTAGAACGCA<br>GCTGCCCCTGAGCCACAACG	First PCR	391	*GGTA1*	[19]
Ex4-2S<br>Ex4-2RV	CTCCTTAGCGCTCGTTGGCT<br>GCAACTCTCTGGAATGCTTT	Nested PCR	351	*GGTA1*	[19]
E-S	AGATATTGGTATAAGCACTTC	Direct sequencing	-	*GGTA1*	[43]
PP-3S<br>PP-3RV	TCCAGAGGCCGCCGGAGG<br>TACTCCCACGTTATAAGA	First PCR	440	*PTEN*	This study[1]
PP-5S<br>PP-OA	ATTATTCGTCTTCTCCCCATT<br>ATGGATACAGGTCAAGTCTA	Nested PCR	347	*PTEN*	This study[1]
PP-6S	GGGCATCAGCTACCGCCAAGT	Direct sequencing	-	*PTEN*	This study[1]
p53-3S<br>p53-3RV	CTGGCATGTGAAGGGCAG<br>CTACTGGGGTTGGGGGCT	First PCR	317	*p53*	This study[2]
p53-4S<br>p53-4RV	TGCCCCAGCAGCAGAGCC<br>AGCAAGGTTATTAGAAGA	Nested PCR	258	*p53*	This study[2]
p53-2S	TCCCCACAGCGGCCACAC	Direct sequencing	-	*p53*	This study[2]
LDLR-S<br>LDLR-RV	AAACCTCACATTGAAATGCTG<br>CCTAAACTCTCGCGCCCCCCT	First PCR	393	*LDLR*	This study[3]
LDLR-2S<br>LDLR-2RV	CTGCAAATGACTGGGGCCCCG<br>CTCCAACCACGTAAGAATGAC	Nested PCR	355	*LDLR*	This study[3]
LDLR-3S	CGCGCGAAGCCCGGATTTCAA	Direct sequencing	-	*LDLR*	This study[3]

[1]Primers are designed based on *Sus scrofa* breed mixed chromosome 14, *Sscrofa* 10.2; NC_010456.4. [2]Primers are designed based on *Sus scrofa* breed mixed chromosome 12, *Sscrofa* 10.2; NC_010454.3. [3]Primers are designed based on *Sus scrofa* breed mixed chromosome 2, *Sscrofa* 10.2; NC_010444.3.

Supplementary Table 2. Summary of the *in vitro* development of SCNT embryos derived from PPA-1.

Treatment with valploic acid (VPA)[1]	No. embryos cultured	No. 2-cell embryos developed (%)	No. blastocysts developed (%)
No	81	52 (64.2)	21 (25.9)
Yes	80	59 (73.8)	25 (31.3)

[1] Treatment of SCNT embryos with 8 mM VPA for 24 h after activation was performed, according to the paper of Miyoshi et al. [45]. Experiments were repeated thrice.

Supplementary Table 3. Primer sets used for PCR for off-target analysis.

Primer sets[1]	Sequence (5′-3′)	Expected size (bp)	Target sequence
For 1st PCR			#1 LDLR (see Supplementary Table 4)
1LDLR-S	TGG AGT GAG GGC GTG TTC TTT		
1LDLR-RV	CCC AGG AGA CAA GAG TCT CAG		
For nested PCR			
1LDLR-2S	CCC ATT AGC AAC TGG GGA GGG	440	
1LDLR-2RV	GGC TCT GCT CCA GCT GAG CCT		
For 1st PCR			#2 LDLR (see Supplementary Table 4)
2LDLR-S	TTT GCT ATT TCT TGG TCT TGG		
2LDLR-RV	AAC CTG CTG AGC TAT GAG GGA		
For nested PCR			
2LDLR-2S	GCC GCT CCC GTG GCA TAT GGA	426	
2LDLR-2RV	ACT CTG GTC CCT CGT TTT CTT		
For 1st PCR			#3 LDLR (see Supplementary Table 4)
3LDLR-S	AAG ATG TAT CTG ACT TCC TTT		
3LDLR-RV	ACC TGT GGC ATG TGG AAG TTC		
For nested PCR			
3LDLR-2S	GGG ATT TAC TTA TAC TCT AAA	434	
3LDLR-2RV	CCA TCC CAG GGA TCA AAC CCA		
For 1st PCR			#1 p53 (see Supplementary Table 4)
1P53-S	GAG CCA TGA TGG GAA CTC CAG		
1P53-RV	TTT ATT GCT TGA GTG TTT CTC		
For nested PCR			
1P53-2S	TTT GCT CTT TGT TGA GTG AAT	419	
1P53-2RV	GCG ATA ACA GCC ATC GGG GAG		
For 1st PCR			#2 p53 (see Supplementary Table 4)
2P53-S	GTG CTG GAA TGT CAT CTC CTG		
2P53-RV	GGG CCA GTA AGT GTT CTC TGA		
For nested PCR			
2P53-2S	TTG CTT CGC TTG GTT AAA TCC	431	
2P53-2RV	CTG GCC GCT TGA TGC CCG CCA		
For 1st PCR			#3 p53 (see Supplementary Table 4)
3P53-S	TTT ATT AAC CAC GTC TCT GTG		
3P53-RV	AGC TCC CGG TTC TCT GTG GTC		
For nested PCR			
3P53-2S	CTG TAG CTC CGT GCC ATG CTG	413	
3P53-2RV	ATT TTC CGA GGC CGC AGC CCT		
For 1st PCR			#1 PTEN (see Supplementary Table 4)
1PT-S	CTT GGC TGC AAA ACG CTG CAC		
1PT-RV	TTG GCC AGA TGC CCA TCT CTC		
For nested PCR			
1PT-2S	AGG GCA CAT GCT ACA CAT TGT	430	
1PT-2RV	TAG AAT CTG TTA ACT TGC TGA		
For 1st PCR			#2 PTEN (see Supplementary Table 4)
2PT-S	GTA CGC TAG GAA AGT GGT GCT		
2PT-RV	AAA CCT GTG GTT ATA AGA AAC		
For nested PCR			
2PT-2S	AGT GGT GCT CCT CAG AAA TCC	444	
2PT-2RV	AGA AAC TCA CTT ATA CCA GTT		
For 1st PCR			#3 PTEN (see Supplementary Table 4)
3PT-S	TTA ACT ATC TGT GCC ACA GGG		
3PT-RV	TTT CAA CTC AAA GGA CAA TTA		
For nested PCR			
3PT-2S	TTA AAG ATG TCT CAG TAG AGC	387	
3PT-2RV	CAA TTG GGT GAG TCA AAC AAA		

[1] PCR was performed twice (1st and nested PCR) to obtain PCR products which will be subjected to a T7E1 assay.

Supplementary Table 4. List of candidate genes that potentially cause off-target mutations.

	Target sequence	Score	Mismatches	Locus
#1 LDLR:	GGAAGGAGGCATGAAGTCCATGG [1]	2.4	3MMs [3:4:8]	chr13:-77692404
#2 LDLR:	AGCTGGAAGCATGGAGTCCAAAG [2]	1.6	2MMs [1:14]	chr5:-9433584
#3 LDLR:	GTCTGTAGGCATGAAGTCCAGAG [3]	1.5	3MMs [2:6:8]	chr5:-51618960

[1]The gRNA (GGCTGGAAGCATGAAGTCCA) for *LDLR* used here exhibits similarity to *Sus scrofa* chromosome 13 clone CH242-223L17, WORKING DRAFT SEQUENCE, 4 unordered pieces Sequence ID: CU633560.2, as shown below.

```
Query  1       GGAAGGAGGCATGAAGTCCATGG  23
               |||||||||||||||||||||||
Sbjct  182529  GGAAGGAGGCATGAAGTCCATGG  182551
```

[2]The gRNA (GGCTGGAAGCATGAAGTCCA) for *LDLR* used here also exhibits similarity to *Sus scrofa* chromosome 5 clone CH242-222P18, WORKING DRAFT SEQUENCE, 5 unordered pieces Sequence ID: CU457758.3, as shown below.

```
Query  1      AGCTGGAAGCATGGAGTCCAAAG  23
              |||||||||||||||||||||||
Sbjct 131570 AGCTGGAAGCATGGAGTCCAAAG  131548
```

[3]The gRNA (GGCTGGAAGCATGAAGTCCA) for *LDLR* used here also exhibits similarity to *Sus scrofa* chromosome 5 clone CH242-507E14, WORKING DRAFT SEQUENCE, 9 unordered pieces Sequence ID: CU582865.2, as shown below.

```
Query  1     GTCTGTAGGCATGAAGTCCAGAG  23
             |||||||||||||||||||||||
Sbjct 93430  GTCTGTAGGCATGAAGTCCAGAG  93452
```

	Target sequence	Score	Mismatches	Locus
#1 p53:	GGAGGAATCGCAGTCCCAGCGGG [1]	2.3	2MMs [7:17]	chr1:-9154440
#2 p53:	GGAGGAGGCACAGTCCGAGGCAG [2]	1.1	3MMs [8:10:20]	chr10:+54637828
#3 p53:	GGAAAAGTCGCATTCCGAGCAGG [3]	1.1	3MMs [4:5:13]	chr6:-4664593

[1]The gRNA (GGAGGAGTCGCAGTCCGAGC) for *p53* used here exhibits similarity to *Sus scrofa* chromosome 1 clone CH242-205C17, WORKING DRAFT SEQUENCE, 7 unordered pieces Sequence ID: FP017297.2, as shown below.

```
Query  1     GGAGGAATCGCAGTCCCAGCGGG  23

             |||||||||||||||||||||||

Sbjct 48297  GGAGGAATCGCAGTCCCAGCGGG  48275
```

[2]The gRNA (GGAGGAGTCGCAGTCCGAGC) for *p53* used here also exhibits similarity to *Sus scrofa* chromosome 10 clone CH242-56F1, WORKING DRAFT SEQUENCE, 4 unordered pieces Sequence ID: CU929835.2, as shown below.

```
Query  1      GGAGGAGGCACAGTCCGAGGCAG  23

              |||||||||||||||||||||||
```

```
Sbjct 129076  GGAGGAGGCACAGTCCGAGGCAG  129098
```

[3]The gRNA(GGAGGAGTCGCAGTCCGAGC) for *p53* used here also exhibits similarity to *Sus scrofa* chromosome 6 clone CH242-270I23, WORKING DRAFT SEQUENCE, 2 unordered pieces Sequence ID: CU929750.2, as shown below.

```
Query  1      GGAAAAGTCGCATTCCGAGCAGG  23
              |||||||||||||||||||||||
Sbjct 102779  GGAAAAGTCGCATTCCGAGCAGG  102801
```

Target sequence		Score	Mismatches	Locus
#1 PTEN:	AGAACTTTAGCAGAAACAAAGGG [1]	3.3	2MMs [4:6]	chr14:-30783590
#2 PTEN:	AGATAGCAAGCAGAAACAAAGAG [2]	1.6	3MMs [5:7:8]	chr11:+42428743
#3 PTEN:	AGAAAATTAGCAGAAACAAAAAG [3]	1.5	3MMs [4:5:6]	chr13:-104741013

[1]The gRNA (AGATCGTTAGCAGAAACAAA) for *PTEN* used here exhibits similarity to *Sus scrofa* chromosome 14 clone CH242-254G23, WORKING DRAFT SEQUENCE, 3 unordered pieces Sequence ID: CU638677.2, as shown below.

```
Query  1     AGAACTTTAGCAGAAACAAAGGG  23
             |||||||||||||||||||||||
Sbjct 63363  AGAACTTTAGCAGAAACAAAGGG  63341
```

[2]The gRNA (AGATCGTTAGCAGAAACAAA) for *PTEN* used here also exhibits similarity to *Sus scrofa* chromosome 11 clone CH242-486J15, WORKING DRAFT SEQUENCE, 18 unordered pieces Sequence ID: CU861566.2, as shown below.

```
Query  1      AGATAGCAAGCAGAAACAAAGAG  23
              |||||||||||||||||||||||
Sbjct 104067  AGATAGCAAGCAGAAACAAAGAG  104045
```

[3]The gRNA (AGATCGTTAGCAGAAACAAA) for *PTEN* used here also exhibits similarity to *Sus scrofa* chromosome 13 clone CH242-13B18, WORKING DRAFT SEQUENCE, 2 unordered pieces Sequence ID: FP236261.2, as shown below.

```
Query  1     AGAAAATTAGCAGAAACAAAAAG  23
             |||||||||||||||||||||||
Sbjct 89317  AGAAAATTAGCAGAAACAAAAAG  89339
```
